# A Comprehensive Evaluation of Possible RNSS Signals in the S-Band for the KPS

**DOI:** 10.3390/s22062180

**Published:** 2022-03-10

**Authors:** Kahee Han, Sanguk Lee, Moonhee You, Jong-Hoon Won

**Affiliations:** 1 Department of Electrical and Computer Engineering, Inha University, Incheon 22212, Korea; kahee.han@inha.edu; 2 KPS Satellite Navigation Research Center, Electronics and Telecommunication Research Institute (ETRI), Daejeon 34129, Korea; slee@etri.re.kr (S.L.); moon@etri.re.kr (M.Y.)

**Keywords:** RNSS, GNSS, KPS, S-band, satellite navigation, modulation

## Abstract

Recently, the Korean government has announced a plan to develop a satellite-based navigation system called the Korean Positioning System (KPS). When designing a new Radio Navigation Satellite Service (RNSS) signal, the use of the S-band has emerged as an alternative to avoiding signal congestion in the L-bands, and South Korea is considering using the S-band with the L-bands. Therefore, this study proposed possible S-band signal candidates and evaluated their performance, such as the radio frequency (RF) compatibility, spectral efficiency, ranging performance, and receiver complexity. Several figures-of-merit (FoMs) were introduced for quantitative performance evaluation for each candidate. Each FoM was calculated using an analytical equation by considering the signal design parameters, such as the center frequency, modulation scheme, and chip rate. The results showed that the outstanding candidate signal was different depending on the signal performance of interest and the reception environments. Therefore, we discuss and summarize the signal performance analysis results considering the whole FoMs together. Under the assumptions given in this paper, the binary phase shift keying (BPSK)(1), sine-phased binary offset carrier (BOC_s_)(5,2), and BPSK signals were superior for the spectral efficiency, ranging performance, and receiver complexity, respectively.

## 1. Introduction

As the importance of satellite navigation systems is gradually increasing, several space powers are building their satellite navigation system or modernizing the existing system to improve its performance. The USA and Russia, pioneers of satellite-based positioning technology, are modernizing their Global Navigation Satellite Systems (GNSSs), called the Global Positioning System (GPS) and the Global Navigation Satellite System (GLONASS), respectively. China has established a three-stage gradual development plan for the BeiDou Navigation Satellite System (BDS) development [1], and in August 2020, officially announced the completion of the deployment of BDS-3 with global coverage [2]. Europe aims to complete the construction of the Galileo system with global coverage by the end of 2023 [3] and plans to launch second-generation Galileo satellites in 2024 [4]. Japan and India are operating regional satellite navigation systems, the Quasi-Zenith Satellite System (QZSS), and the Navigation with Indian Constellation (NavIC). Currently, these regional systems are composed of four and seven satellites, respectively, and they plan to launch additional satellites [5,6]. Recently, the government of South Korea officially announced the development of its satellite-based positioning system, called the Korean Positioning System (KPS), to provide services from 2035 [7,8,9].

Satellite-based navigation systems can use only the frequency band allocated to the Radio Determination Satellite Service (RDSS) or Radio Navigation Satellite Service (RNSS) regulated by the International Telecommunication Union (ITU). According to the ITU Radio Regulations, downlink satellite navigation signals can use the L-band (1164–1300 MHz, 1559–1610 MHz), S-band (2483.5–2500 MHz), and C-band (5010–5030 MHz). Traditionally, the L-band has been preferred for satellite navigation systems. However, the number of signals located in the L-band is increasing rapidly, many researchers have studied the utilization and availability of the S-band or C-band for new RNSS signals.

The study on the use of the C-band for RNSS signals began in earnest by adopting Resolution 604 at the World Radiocommunication Conference (WRC) held in 2000. The resolution allows for the transmission of navigation signals from the RNSS space station to Earth over the 5010–5030 MHz band. Europe considered the use of the C-band in the early phase of Galileo development. However, due to the technical limitations at that time, the C-band was excluded from the first generation of Galileo [10]. Since then, the use of the C-band was reconsidered for a future generation of Galileo development [11,12,13,14]. According to the previous studies, the use of the C-band causes high free space loss due to the use of frequencies that are three to four times higher than that of the L-band. The C-band also has drawbacks, such as high phase noise and high signal attenuation due to atmosphere and obstacles compared to the L-band. Due to these drawbacks, no RNSS signal is being transmitted through the C-band.

At the World Administrative Radio Conference for the Mobile Services held in 1987 (WARC MOB-87), the S-band was additionally allocated to the RDSS on a primary or secondary basis in some countries [15,16]. After WRC-12, this band was allocated to RDSS on a worldwide primary basis. The use of the S-band also causes high free space loss compared to the L-band but less than that of the C-band. In addition, the payload implementation is simple than when using the C-band. For this reason, South Korea is researching the use of the S-band together with the L-bands. Europe and China are also investigating the use of the S-band for the next generation Galileo and BDS [17,18,19,20,21,22,23].

However, most previous research on the use of the S-band was conducted on new modulation schemes, such as continuous phase modulation (CPM) or orthogonal frequency division multiplexing (OFDM) [17,18]. The CPM and OFDM schemes provide high spectral efficiency. However, they have several drawbacks, such as requiring complex decoders, being highly sensitive to frequency offsets, and so on [19,20]. Therefore, traditional modulation schemes, such as binary phase-shift keying (BPSK) and binary offset carrier (BOC) modulation, are still attractive signal design options. BPSK and BOC signals were analyzed as new RNSS signal candidates in the S-band [21,22,23], but only a few scenarios were considered in the previous studies.

The main contribution of this study was to comprehensively evaluate the navigation signal performance of possible BPSK and BOC signals using figures-of-merit (FoMs). The FoMs were determined using RNSS signal design parameters, such as the modulation scheme, chip rate, subcarrier frequency, and so on [24]. Therefore, we clarify the relationship between each FoM and the signal design parameters and analyze the FoMs for each candidate. Since the FoMs have a trade-off relationship [25], we distinguished suitable candidates as S-band signals for KPS by considering the FoMs analysis results together.

The rest of this paper consists of the following. Section 2 describes a radio frequency (RF) system using the S-band and adjacent bands. Section 3 introduces several FoMs that quantify navigation signal performance, and then Section 4 provides FoM analysis results for new RNSS signal design candidates. Finally, we discuss the FoM analysis results and findings in Section 5 and conclude the paper in Section 6.

## 2. S-Band and Adjacent Bands Systems

When designing a new navigation signal, investigations on the existing systems in the same and adjacent bands should be undertaken to analyze RF compatibility. Services using the S-band and adjacent bands via ITU frequency allocation are listed in Table 1. The S-band is shared with several primary services, including RDSS, and various terrestrial wireless communication services are being operated in the adjacent bands [26].

### 2.1. Navigation with Indian Constellation

India’s NavIC is a regional RDSS system consisting of three geostationary Earth orbits (GEOs) and two inclined geosynchronous orbits (IGSOs) inclined at 29° from the equatorial plane. There are three satellites in GEO (at 32.5° E, 83° E, and 131.5° E) and two satellites in each IGSO (with longitude crossing at 55° E and 111.75° E). The primary service area includes the region extending up to 1500 km from the Indian border. NavIC plans to deploy four additional IGSO satellites to improve the service availability, accuracy, and continuity and expand the coverage [27].

Currently, NavIC is the only satellite navigation system that transmits a navigation signal in the S-band, providing a standard positioning service (SPS) and a restricted service (RS) [28]. The carrier frequency of NavIC in the S-band is at 2492.028 MHz, and the design parameters for these signals are given in Table 2.

### 2.2. Globalstar

Globalstar is a mobile satellite service (MSS) system that provides mobile voice and data communication to global users. The system consists of 48 low Earth orbit (LEO) satellites at 1,414 km altitude and terrestrial gateways. This system divides the bandwidth of 16.5 MHz into 13 frequency division multiplexing (FDM) channels with a bandwidth of 1.23 MHz [29,30]. A code division multiple access (CDMA) technique is used for each FDM channel, and the spreading sequence consists of inner and outer pseudorandom noise (PN) sequences. The chip rate and length are 1.2288 Mcps and 1024 chips for the inner PN sequence and 1.2 kcps and 288 chips for the outer PN sequence [30].

The baseband signal is filtered by the Nyquist square-root-raised-cosine (SRRC) filter with a roll-off factor of 0.2, and the power spectral density (PSD) of the SRRC filter applied to the kth FDM channel can be expressed as follows [21]:

(1) $$G_{SRRC}^{k}\left(f\right)=\{\begin{array}{ccc} 1 & if & \left|f-kB\right|\leq \frac{B}{2}\left(1-\rho \right)\\ 0 & if & \left|f-kB\right|\geq \frac{B}{2}\left(1+\rho \right)\\ g\left(f\right) & if & \frac{B}{2}\left(1-\rho \right)\leq \left|f-kB\right|\leq \frac{B}{2}\left(1+\rho \right) \end{array}$$

with

(2) $$g\left(f\right)=\frac{1}{2}+\frac{1}{2}\cos\left(\frac{\pi }{2\rho f_{c}}\left(f-\left(1-\rho \right)f_{c}-kB\right)\right)$$

where k is an FDM channel index (k=−6,−5,…, 6), ρ is a roll-off factor, B is a bandwidth of a single FDM channel, and $f_{c}$ is a cutoff frequency (same as B/2).

### 2.3. Other Communication Systems in Adjacent Bands

The industrial, scientific, and medical (ISM) band and long-term evolution (LTE) band are located near the S-band. According to article No.5.150 in [26], the ITU allocates some frequency bands, including the 2400–2500 MHz band, for ISM applications. Since ISM bands are unlicensed bands, short-range devices (SRDs) providing low-power/short-range radiocommunication also operate in ISM bands [31]. SRD applications include wireless-fidelity (Wi-Fi), Bluetooth, and near-field communication (NFC) [32]. For the 2.4 GHz ISM band, only the band 2400–2483.5 MHz can be used for SRD applications in North America, Europe, and South Korea [33].

**Figure 1:**
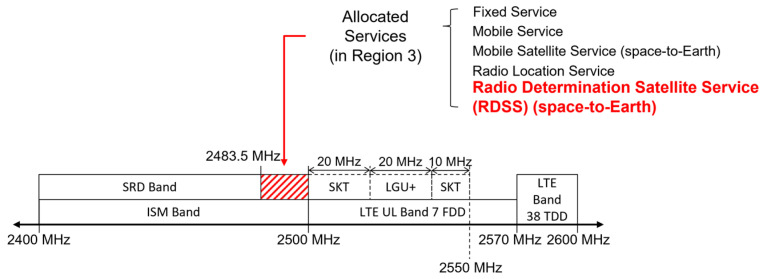
Frequency allocation of the 2.4–2.6 GHz band in South Korea.

The band from 2500 MHz to 2570 MHz is allocated to the LTE band 7 uplink by the ITU. In South Korea, the leading mobile operators, namely, SK Telecom and LG U+, use the band 2500–2550 MHz. Figure 1 shows the frequency allocation from 2.4 GHz to 2.6 GHz in South Korea. Most radio devices equipped with RNSS receivers also provide short-range and/or LTE radiocommunication functions. Considering that there is an in-device coexistence interference problem between Bluetooth, Wi-Fi, and LTE [34], we can anticipate in-device coexistence interference between RNSS and the adjacent bands’ systems.

## 3. Navigation Signal Design Considerations

One of the main challenges in RNSS signal design is that the payload power source and frequency resource are limited. Additionally, a new RNSS signal should guarantee compatibility with the existing RNSS and other systems. In other words, when designing a new RNSS signal, we have to consider ranging performance, power/frequency constraints, and compatibility with existing systems.

However, it is not easy to evaluate these considerations through the practical RNSS signal transmission/reception chain at the initial signal design phase. Therefore, we conducted a performance evaluation and analysis on the signal candidates based on various FoMs, defined as a function of the signal design parameters, which enabled quantitative performance evaluation and comparison between the candidates [24]. This study focused on several signal design considerations, such as RF compatibility, spectral efficiency, ranging performance, and receiver complexity.

### 3.1. RF Compatibility

When designing a new RNSS signal, RF compatibility analysis between existing signals sharing the desired frequency band and candidate signals is essential. For example, RF compatibility with GPS was a key driver for determining the Galileo signal plan [35].

The interference analysis methodology described in ITU-R M.1831 is used for RF compatibility analysis between RNSS systems [36]. This methodology can also be applied to RF compatibility analysis with Globalstar [21]. This methodology uses the effective carrier-to-noise density ratio, i.e., effective $C/N_{0}$, as a measure of the impact of the interference from various interferers on the desired system. The effective $C/N_{0}$ is obtained by modeling the interference as a white noise that induces the same performance degradation on the correlator output as follows [36]:

(3) $${\left(C/N_{0}\right)}_{eff}=\frac{C}{N_{0}+I_{intra}+I_{inter}+I_{ext}}$$

with

(4) $$I_{x}=\overset{N}{\underset{i=1}{\sum }}C_{i}\frac{\int_{-B_{r}/2}^{B_{r}/2}G_{s}\left(f\right)G_{i}\left(f\right)df}{\int_{-B_{r}/2}^{B_{r}/2}G_{s}\left(f\right)df}$$

where *C* is the received power of the desired signal, *N*_0_ is the noise power density, and *I_x_* (*I_intra_*, *I_inter_*, *I_ext_*) are the equivalent noise power density of intrasystem, intersystem, and external system interference, respectively. Assuming that each system consists of *N* satellites, the equivalent noise power density *I_x_* (*I_intra_*, *I_inter_*, or *I_ext_*) is calculated using Equation (4), where *C_i_* is the received power of the interfering signals from the *i*th satellite, *B_r_* is the receiver front-end filter bandwidth, and *G_s_*(*f*) and *G_i_*(*f*) are the normalized PSD to unity over the transmission bandwidth of the desired and interfering signals, respectively.

The received power of the interference signal depends on the distance between the receiver and visible satellite, the number of visible satellites, transmitted power, and antenna gain at the elevation angle and off-boresight angle. However, these factors are time-varying and not related to the signal waveform. Therefore, when we design a signal waveform, we analyze RF compatibility by considering only the PSD of the desired and interfering signals independently from the above factors using the spectral separation coefficient (SSC) [37]. SSC is defined as shown in Equation (5), where a higher SSC lets the receiver undergo severe interference at the same received interfering signal power.

(5) $$k_{is}=\int_{-B_{r}/2}^{B_{r}/2}G_{s}\left(f\right)G_{i}\left(f\right)df$$

### 3.2. Spectral Efficiency

The RF system can only use the frequency band allocated by ITU and should not cause harmful interference to services that operate in adjacent bands. In order to suppress out-of-band emission (OOBE), frequency components outside the allocated frequency band must be filtered at the transmitter. Therefore, we should concentrate signal power within the transmission bandwidth to minimize signal distortion due to filtering and effectively use limited transmit power.

In order to maintain the signal power before the filtering, the signal should be amplified by the factor $\lambda_{OOBE}$ to compensate for the filtering loss. The $\lambda_{OOBE}$ value refers to a ratio of signal power distributed to the out-of-band emission. The factor is expressed as [38]:

(6) $$\lambda_{OOBE}=\frac{1}{\int_{-B/2}^{B/2}G_{s}\left(f\right)df}=\frac{1}{\eta }$$

where *B* is the allocated bandwidth (or transmit bandwidth) and *G_s_*(*f*) is the PSD normalized in an infinite bandwidth. The reciprocal of $\lambda_{OOBE}$ is the ratio of signal power distributed within the allocated frequency band and it refers to spectral efficiency *η*.

### 3.3. Ranging Performance

The purpose of the RNSS signal is to provide accurate ranging information to the user. The position accuracy at a receiver is determined by the pseudorange error and the user-satellite geometry. The pseudorange error can be modeled as a user equivalent range error (UERE) budget consisting of a delay-locked-loop (DLL) error, multipath error, ionosphere/tropospheric errors, satellite clock/orbit errors, and so on [39]. Among them, the DLL error and the multipath error depend on the signal modulation scheme.

#### 3.3.1. DLL Error

The DLL error is a component of the UERE budget and depends on the signal reception environment (e.g., $C/N_{0}$) and the signal waveform. The DLL error refers to inherent code tracking error due to signal waveform and can be analyzed in terms of accuracy and stability. Assuming only white noise exists, the 1 sigma value of the DLL error *σ_DLL_* is determined by the thermal noise $\sigma_{tDLL}$ and the dynamic stress error $R_{e}$.

(7) $$\sigma_{DLL}=\sigma_{tDLL}+\frac{R_{e}}{3}$$

For the stable DLL tracking in the receiver, the value of *σ_DLL_* should not exceed one-sixth of the linear pull-in range of the DLL discriminator. Typically, the pull-in range of the DLL discriminator is defined as ±d/2, where d is the correlator spacing in chips [40].

The dynamic stress error depends on the loop order and user dynamics. Therefore, we consider only the thermal noise jitter to evaluate the DLL error for signal design candidates. The thermal noise jitter is determined according to the DLL discriminator type. When using coherent early-minus-late processing (CELP) and noncoherent early-minus-late processing (NELP) as discriminators, the DLL thermal noise jitter (in seconds) is defined as follows [41,42,43]:

(8) $$\sigma_{tDLL,CELP}^{2}=\frac{B_{L}\int_{-B_{r}/2}^{B_{r}/2}G_{s}\left(f\right)\sin^{2}\left(\pi fd\right)df}{{\left(2\pi \right)}^{2}\frac{C}{N_{0}}{\left(\int_{\frac{-B_{r}}{2}}^{\frac{B_{r}}{2}}fG_{s}\left(f\right)\sin\left(\pi fd\right)df\right)}^{2}}$$

(9) $$\sigma_{tDLL,NELP}^{2}=\frac{B_{L}\int_{-B_{r}/2}^{B_{r}/2}G_{s}\left(f\right)\sin^{2}\left(\pi fd\right)df}{{\left(2\pi \right)}^{2}\frac{C}{N_{0}}{\left(\int_{\frac{-B_{r}}{2}}^{\frac{B_{r}}{2}}fG_{s}\left(f\right)\sin\left(\pi fd\right)df\right)}^{2}}\left[1+\frac{\int_{-B_{r}/2}^{B_{r}/2}G_{s}\left(f\right)\cos^{2}\left(\pi fd\right)df}{T\frac{C}{N_{0}}{\left(\int_{\frac{-B_{r}}{2}}^{\frac{B_{r}}{2}}G_{s}\left(f\right)\cos\left(\pi fd\right)df\right)}^{2}}\right],$$

where $B_{L}$ is the DLL bandwidth and T is the coherent integration time.

#### 3.3.2. Gabor Bandwidth

As shown in Equations (8) and (9), the DLL thermal noise jitter depends on $C/N_{0}$ and the receiver parameters, such as discriminator type, DLL bandwidth, coherent integration time, and correlator spacing. The Gabor bandwidth (so-called root mean square (RMS) bandwidth) is used to evaluate the code tracking performance of RNSS signals independent of the receiver parameters above. The Gabor bandwidth is derived from the Cramer–Rao lower bound (CRLB) of the DLL thermal noise jitter, where the CRLB $\sigma_{CRLB}^{2}$ and the Gabor bandwidth $f_{Gabor}$ are expressed as follows [17]:

(10) $$\sigma_{CRLB}^{2}=\frac{B_{L}}{{\left(2\pi \right)}^{2}\frac{C_{s}}{N_{0}}\int_{\frac{-B_{r}}{2}}^{\frac{B_{r}}{2}}f^{2}G_{s}\left(f\right)df}=\frac{B_{L}}{{\left(2\pi \right)}^{2}\frac{C_{s}}{N_{0}}f_{Gabor}}$$

with

(11) $$f_{Gabor}=\int_{-B_{r}/2}^{B_{r}/2}f^{2}G_{s}\left(f\right)df.$$

From Equation (11), we can expect that the Gabor bandwidth has a higher value when the signal power is more concentrated on the high-frequency components. A higher Gabor bandwidth refers to the signal that has a better code-tracking performance.

#### 3.3.3. Multipath Error

Multipath signals (echoes) of which the delay time $\tau_{M}$ is shorter than $T_{c}\left(1+\frac{d}{2}\right)$ distort the correlation function between the direct signal and the local replica, resulting in a pseudorange error [44], where $T_{c}$ is the chip width. Assuming that the receiver front-end bandwidth is infinite, the correlation function distorted by one multipath signal is expressed as

(12) $$R_{comp}\left(\tau \right)=R\left(\tau \right)+\alpha R\left(\tau -\tau_{M}\right)\cdot \cos\left(\Delta \psi \right)$$

where α is the multipath-to-direct ratio (MDR) and Δψ is the phase difference between the direct and multipath signals.

The distorted correlation function deteriorates the code-tracking performance and its effect depends on the discriminator type. Assuming that the receiver front-end bandwidth is infinite and there exists a single multipath signal, the CELP and NELP discriminator outputs can be expressed as Equations (13) and (14), respectively [45]:

(13) $$\varepsilon_{MP,CELP}\left(\Delta \tau \right)=\left[R\left(\Delta \tau -\frac{d}{2}\right)+\alpha R\left(\Delta \tau -\tau_{M}-\frac{d}{2}\right)\cos\left(\Delta \psi \right)\right]-\left[R\left(\Delta \tau +\frac{d}{2}\right)+\alpha R\left(\Delta \tau -\tau_{M}+\frac{d}{2}\right)\cos\left(\Delta \psi \right)\right]$$

(14) $$\varepsilon_{MP,NELP}\left(\Delta \tau \right)={\left[R\left(\Delta \tau -\frac{d}{2}\right)+\alpha R\left(\Delta \tau -\tau_{M}-\frac{d}{2}\right)\cos\left(\Delta \psi \right)\right]}^{2}-{\left[R\left(\Delta \tau +\frac{d}{2}\right)+\alpha R\left(\Delta \tau -\tau_{M}+\frac{d}{2}\right)\cos\left(\Delta \psi \right)\right]}^{2}$$

where Δτ is the code tracking error in chips.

### 3.4. Receiver Complexity

The correlation result between the received signal and the local replica of the desired signal is used in the acquisition and tracking processes. Assuming that there is no Doppler effect and the spreading code length is infinite, the autocorrelation function of BPSK signals does not have a sidelobe peak. However, in the case of BOC signals, it has significant sidelobe peaks. These non-negligible sidelobe peaks can cause false detection and false locks in the low signal-to-noise ratio (SNR) [42]. The implementation complexity of algorithms proposed to avoid the false detection and lock problem depends on the shape of the autocorrelation function of the desired signal [46,47,48,49]. The higher the sidelobe peaks, the more complex algorithms are required.

The autocorrelation main peak–to–secondary peak ratio (AMSR) is the ratio of the second-highest peak value to the highest peak value of the autocorrelation function. The AMSR is defined as

(15) $$AMSR=\frac{max\left\{R^{2}\left(\tau \right)\right\}}{R^{2}\left(0\right)},\;\tau \notin Mainlobe$$

where *R*(*·*) is the normalized autocorrelation function with a main peak value of 1 and *τ* is the code offset between the received signal and the local replica.

## 4. FoMs Analysis

In previous research, BPSK(1), BPSK(4), BPSK(8), BOC_s_(1,1), BOC_c_(4,4), BOC_s_(4,4), and multiplexed BOC (MBOC) were studied as future S-band navigation signals [17,22]. Meanwhile, one of the concerns regarding using the S-band is the OOBE interference due to the adjacent band systems [31,32,50,51]. If the RNSS signal has a large amount of signal power near the edge of the S-band, more serious interference is expected. Therefore, we considered additional candidates that have less power at the edge of the frequency band.

Furthermore, the cosine-phased BOC modulation with the same chip rate and subcarrier frequency of the sine-phased BOC scenarios were examined as candidates. BPSK(1) and BOC_s_(5,2), which are adopted modulation schemes in the NavIC S-band signals, were also considered. Table 3 specifies the carrier frequency and modulation schemes of the candidate signals investigated in this study.

### 4.1. SSC Analysis Results

For RF compatibility analysis in the S-band, we considered existing RNSS and MSS systems (i.e., NavIC and Globalstar). RF compatibility between the candidate signals and the existing signals was evaluated using the SSC.

Even when the modulation scheme is the same, the SSC can vary with the front-end bandwidth of the receiver. The front-end bandwidth of the receiver is determined by the receiver’s uses. Receivers for precise positioning use, such as geodetic surveys, have a wide bandwidth. However, low-cost receivers, such as RNSS receivers embedded in smartphones, have a narrow bandwidth to reduce the implementation cost and front-end size.

Therefore, we analyzed the SSC, assuming a front-end bandwidth of 16.5 MHz or the same as the main lobe width of the candidate signal. In the latter case, the front-end bandwidth is defined as 2*f_c_* for BPSK signals and 2(*f_s_* + *f_c_*) for BOC signals. Table 4 enumerates the SSC between the candidate signals and the existing signals in the S-band.

Since the NavIC SPS signal has a narrow main lobe, it has a low SSC for BOC signals. BOC_c_(4,4) had the lowest SSCs with the NavIC SPS signal as −85.97 for both front-end bandwidths. If the chip rate and subcarrier frequency are the same, the cosine-BOC signal has a lower SSC than the sine-BOC signal. This is because the shape of the PSD of the cosine-phased BOC has smaller sidelobes between the two main lobes than sine-phase BOC. This characteristic was considered in the case of changing the Galileo E6 Public Regulated Service (PRS) signal from BOC_s_(10,5) to BOC_c_(10,5) to increase the spectral separation with the E6 Commercial Service (CS) signal.

**Figure 2:**
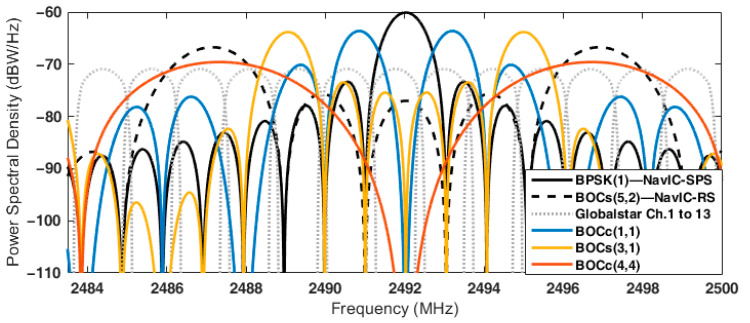
Normalized power spectral density (PSD) of NavIC, Globalstar, and candidate binary phased shift keying (BPSK) and binary offset carrier (BOC) signals in the S-band.

The SSC between the candidates and NavIC RS signal is considerably different according to the receiver’s front-end bandwidth. When the front-end bandwidth is 16.5 MHz, the SSC of BOC_s_(3,1) was the lowest among the candidates at −80.34. However, when the front-end bandwidth was equal to the candidate’s main lobe width, BOC_c_(1,1) had the lowest SSC at −84.27. As shown in Figure 2, this was because the spectral overlap region between the side lobe of BOC_c_(1,1) and the main lobes of the NavIC RS signal was not considered in the SSC calculation when the receiver bandwidth was limited as the main lobe width of the candidate.

Each FDM channel of the Globalstar signal has a narrow bandwidth. Therefore, the wider the main lobe of candidates and the greater the center frequency of the main lobe differs significantly from the center frequency of each FDM channel, the lower the SSC value. Self SSC for evaluating intra-system interference has a lower value as the signal power spreads widely. Among the candidates, BOC_c_(4,4) had the lowest SSC at −72.16 for both front-end bandwidths.

### 4.2. Spectral Efficiency Analysis Results

Considering that the S-band has a narrow bandwidth of 16.5 MHz, the use of a signal with high spectral efficiency is required. Table 5 shows the spectral efficiency of the candidates calculated using Equation (6). Among the BPSK signals, BPSK(1) had the highest spectral efficiency with 0.99, and BPSK(8) had the lowest with 0.91. Among the BOC signals, BOC_s_(1,1) had the highest spectral efficiency with 0.97, and BOC_c_(4,4) had the lowest with 0.75. A common feature of BPSK(8) and BOC_c_(4,4) is that they do not have any side lobes within the allocated S-band. In the case of having the same subcarrier frequency and chip rate, the sine-BOC signal had higher spectral efficiency than the cosine-BOC signal.

### 4.3. DLL Error Analysis Results

The DLL error depends on the receiver parameters, as well as the signal waveforms. Therefore, we analyzed the DLL error using the specified receiver parameters in Table 6.

**Figure 3:**
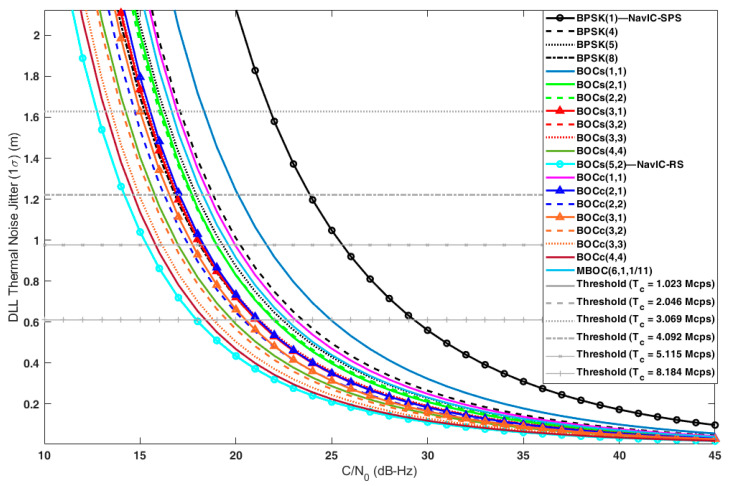
The DLL errors and stability thresholds of the candidates.

Figure 3 shows the DLL thermal noise jitter with respect to $C/N_{0}$. As defined in Equation (10), the DLL error decreased as $C/N_{0}$ increased. DLL stability was analyzed using a 1−σ noise jitter and a stability threshold corresponding to d/6. The threshold was a function of the chip rate when expressed in meters. Therefore, each candidate had different threshold values depending on its chip rate, even though they had the same correlator spacing. The $C/N_{0}$ corresponding to the intersection of the noise jitter curve and the threshold line gave the minimum required $C/N_{0}$ for the DLL to operate in a stable region.

Table 7 summarizes the 1σ DLL thermal noise jitter of each candidate when $C/N_{0}$ was 20 dB-Hz, 40 dB-Hz, and the minimum required $C/N_{0}$ for the stable DLL tracking. From the results, we found two things. First, the higher the chip rate and subcarrier frequency, the better the code tracking performance. Second, if the cosine-BOC signal and the sine-BOC signal had the same chip rate and subcarrier frequency, the cosine-BOC had a better code-tracking performance.

Among the candidates, BOC_s_(5,2) showed the best code-tracking performance in terms of both accuracy and stability. BOC_s_(5,2) had a minimum required $C/N_{0}$ of 10.79 dB-Hz, and when $C/N_{0}$ was 40 dB-Hz, the DLL error was 0.03 m. The minimum required $C/N_{0}$ and the DLL error of BPSK(1), which were the worst candidates in terms of the code-tracking performance, were 4.3 dB and 0.14 m higher than those of BOC_s_(5,2), respectively.

### 4.4. Gabor Bandwidth Analysis Results

**Figure 4:**
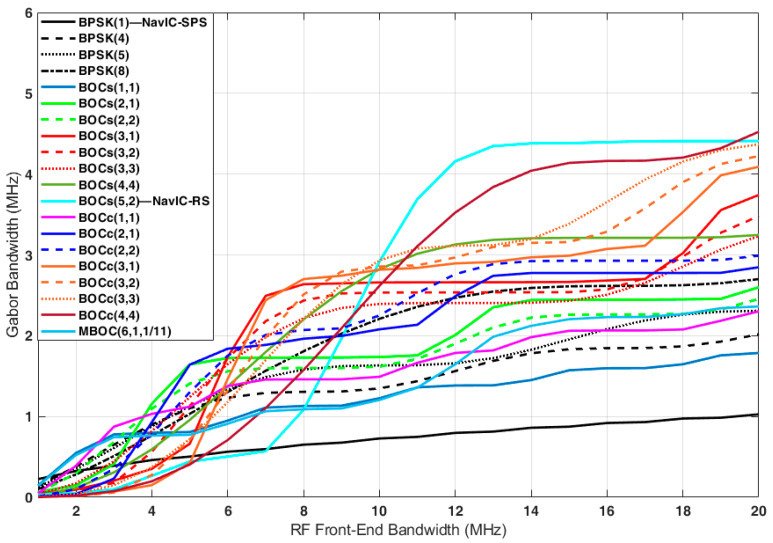
Gabor bandwidth of the candidates in the S-band.

The Gabor bandwidths of each candidate are shown in Figure 4. The Gabor bandwidth increased as the front-end bandwidth increased. For BOC signals, the front-end bandwidth increased steeply at the point where it contained two main lobes of the PSD.

The front-end bandwidth differs depending on the uses of the receiver. Therefore, we analyzed the Gabor bandwidth when the front-end bandwidth was 4 MHz, 16.5 MHz, or corresponded to the main lobe bandwidth. The Gabor bandwidths for each case are listed in Table 8.

When the front-end bandwidth was 4 MHz, BOC_c_(3,1) only had small side lobes within the bandwidth. Therefore, it had the smallest Gabor bandwidth of 0.15 MHz. Among the candidates, BOC_s_(2,1) had the largest Gabor bandwidth of 1.16 MHz. The PSD of BOC_s_(2,1) was maximized at ±2 MHz and had a large side lobe at the center frequency.

When the front-end bandwidth was 16.5 MHz or corresponded to the main lobe width, BPSK(1) and BOC_s_(5,2) had the minimum and maximum Gabor bandwidths, respectively. Similar to the code tracking performance, with the same chip rate and subcarrier frequency, the cosine-BOC signal had a larger Gabor bandwidth than the sine-BOC signal.

### 4.5. Multipath Error Analysis Results

The multipath error is a function of the multipath delay, and the multipath error characteristic of the signal can be analyzed using the multipath error envelope (MPEE). The MPEE is composed of a positive envelope and a negative envelope. These two envelopes indicate the upper and lower bounds of the pseudorange error due to in-phase and quadrature multipaths, respectively [44].

**Figure 5:**
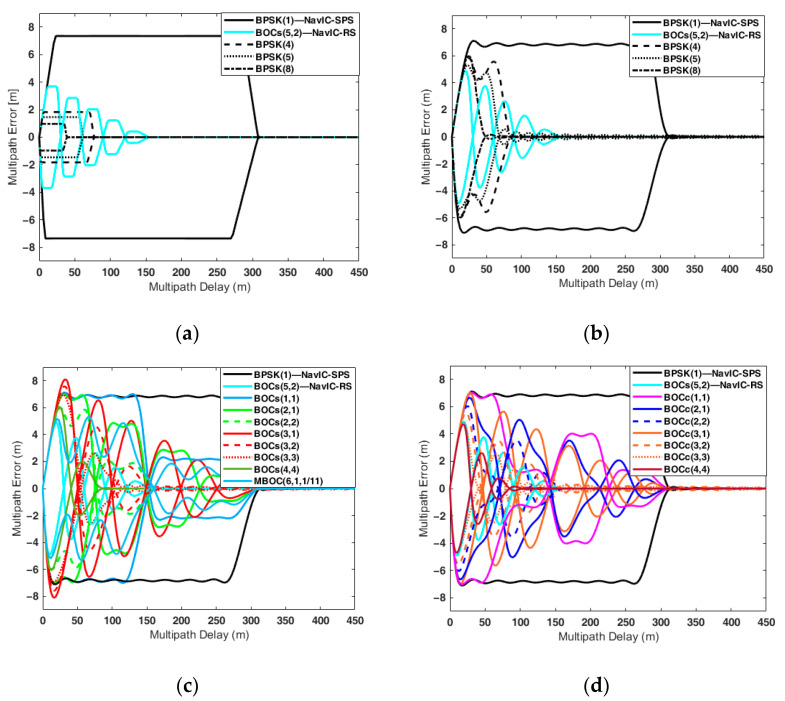
Multipath error envelope of the candidates in the S-band: (**a**) $B_{r}=\infty$; (**b**–**d**) $B_{r}$ = 16.5 MHz.

The multipath error was analyzed using the receiver parameters in Table 6, assuming there existed only one single multipath with the MDR of −3 dB. Figure 5 illustrates the MPEE for all candidate signals, and Table 9 enumerates the maximum multipath error of each candidate.

Figure 5a,b shows the MPEE of the BPSK and NavIC signals. When the receiver bandwidth was infinite (Figure 5a), the higher the chip rate, the smaller the maximum multipath error. Similar to the DLL error, the limited front-end bandwidth affected the multipath error. The autocorrelation function was distorted due to the limited bandwidth, causing the MPEE to fluctuate, and the MPEE fluctuation gave the higher maximum multipath error. The higher the chip rate, the more severe the multipath performance degradation was due to the limited bandwidth (Figure 5b). When the front-end bandwidth was 16.5 MHz, the maximum multipath error of BPSK(8) was 5.98 m, which was about six times larger than the 0.98 m when the front-end bandwidth was infinite.

Figure 5c,d shows the MPEE of the sine-BOC and cosine-BOC candidates when the front-end bandwidth was 16.5 MHz, where it can be seen that the lower the chip rate, the larger the maximum multipath error. BOC_c_(3,3) had the smallest maximum multipath error at 4.8 m, and BOC_s_(3,1) had the largest maximum multipath error at 8.12 m.

On the other hand, it is not intuitive to use the MPEE to evaluate multipath performance. This is because the MPEE is composed of two envelopes, and in some cases, the envelope heavily fluctuates. Therefore, we used a running average (RA) of MPEE to overcome this problem.

**Figure 6:**
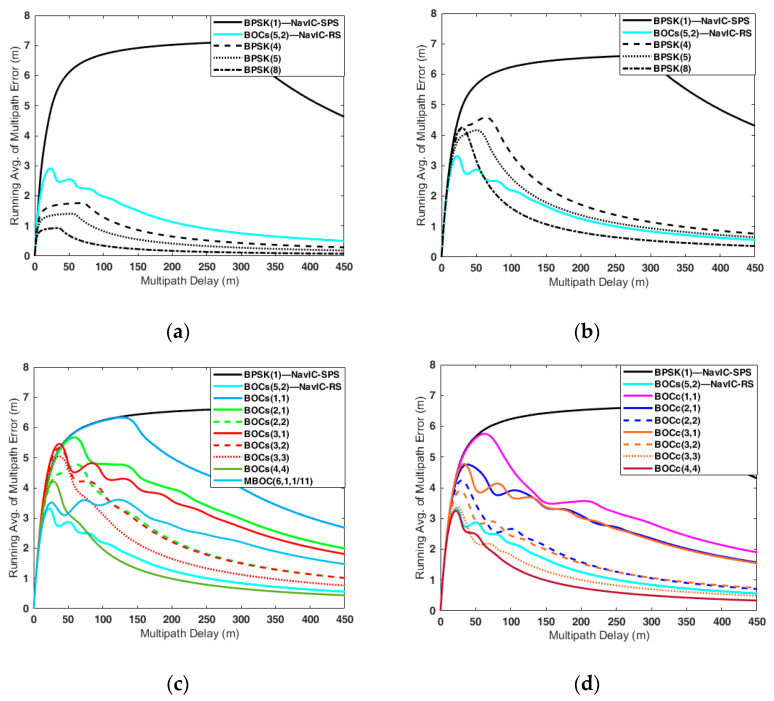
Running average of the multipath error envelope of the candidates in the S-band: (**a**) $B_{r}=\infty$; (**b**–**d**) $B_{r}$ = 16.5 MHz.

The RA of the MPEE can be obtained by using the cumulative sum of the absolute envelope values for the multipath delays [45]. Figure 6 shows the RA of the MPEE corresponding to Figure 5, and Table 9 enumerates the RA values for specific multipath delays reflecting the band-limited effect.

According to [45], the typical multipath delays in open and urban environments are 26 m and 51 m, respectively. When the multipath delay was 26 m, BOC_s_(5,2) had the lowest RA value. This means that BOC_s_(5,2) was the most robust to the multipath in the open environment. In the urban environment, namely, when the multipath delay was 51 m, BOC_c_(3,3) had the best multipath resistance performance. If the multipath delay was more than 62 m, BOC_c_(4,4) had the best multipath performance, and its RA value was 0.37 m with a multipath delay of 400 m.

### 4.6. AMSR Analysis Results

Table 10 enumerates the AMSR of each candidate signal according to the receiver front-end bandwidth. The receiver filter is assumed to be an ideal brick-wall filter with a unity gain over the passband. Note that values less than 0.01 were considered as 0.

**Figure 7:**
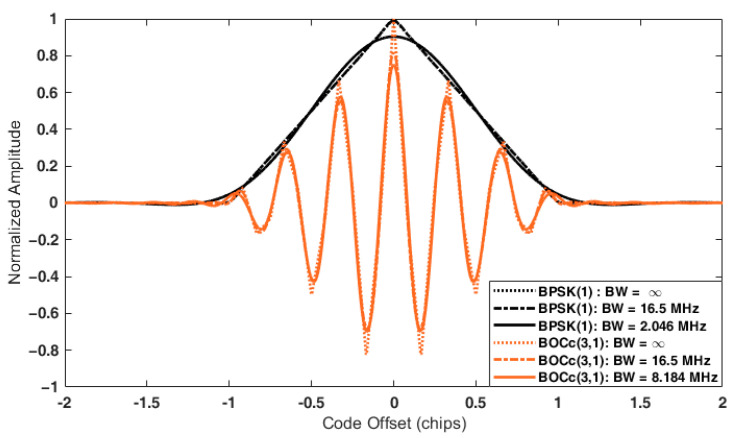
Autocorrelation function of BPSK(1) and BOC_c_(3,1).

If the receiver bandwidth is infinite, the AMSR of the BPSK signal is theoretically zero. The BOC signal has a larger AMSR as the modulation order is higher. The autocorrelation function distorted by the filtering increases AMSR. The cosine-BOC signal was more affected by the filtering due to the shape of the PSD, and the difference in the AMSR value according to the front-end bandwidth was relatively significant. Figure 7 shows the autocorrelation function according to the front-end bandwidth of BPSK(1) with the smallest AMSR and BOC_c_(3,1) with the largest AMSR.

## 5. Discussion

The S-band has emerged as the desirable frequency band for the next-generation RNSS signal. Therefore, South Korea is considering using the S-band for the KPS. For this reason, we investigated possible RNSS signals in the S-band and analyzed their RF compatibility, spectral efficiency, ranging performance, and receiver complexity using the FoMs.

The signal performance of the candidates is evaluated differently by the reception environment. Therefore, this section summarizes and discusses the simulation results under the specific receiver parameters in Table 6 and the multipath delay of 51 m.

**Figure 8:**
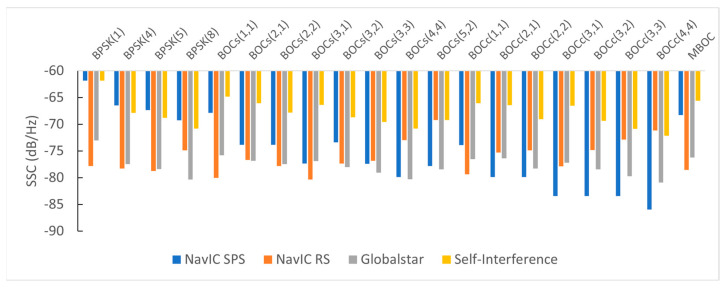
The SSC values between the existing S-band signals and each candidate.

Figure 8 shows the SSCs of the existing S-band signals with each candidate. A low SSC means that the RF compatibility between the two signals was good. The SSCs between candidates and the NavIC SPS signal had a different tendency than SSCs between the NavIC RS signal. In other words, candidates having good RF compatibility with the NavIC SPS signal had poor RF compatibility with the NavIC RS signal. BOC_c_(4,4) and BOC_s_(3,1) had the best RF compatibilities with the NavIC SPS signal and RS signal, respectively. Meanwhile, BOC_c_(4,4) had the best RF compatibilities with the Globalstar and itself.

**Figure 9:**
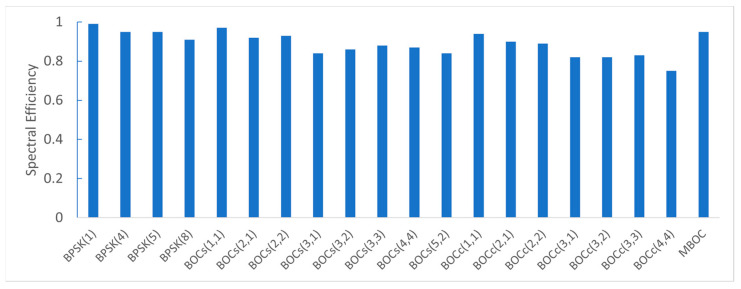
The spectral efficiency for each candidate.

The spectral efficiencies for each candidate are shown in Figure 9. A high spectral efficiency indicated that the signal power distribution was concentrated within 16.5 MHz. BPSK signals had high spectral efficiency and BOC_c_(4,4) had the worst spectral efficiency.

**Figure 10:**
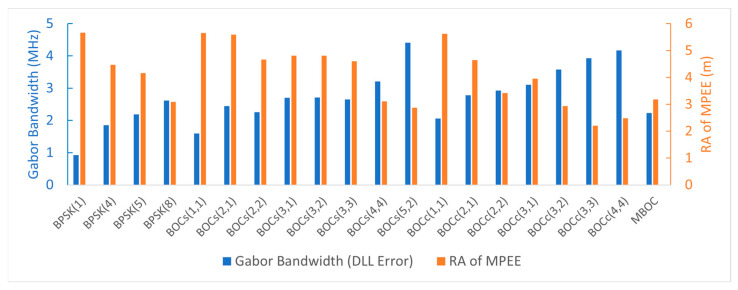
The Gabor bandwidth and the RA of the MPEE for each candidate.

The ranging performance was analyzed using the Gabor bandwidth and the RA of the MPEE. Figure 10 shows the Gabor bandwidth and the RA of the MPEE for each candidate. A high Gabor bandwidth and a small RA of the MPEE imply that the signal has good ranging performance. BOC_s_(5,2) was the best in terms of the Gabor bandwidth (also DLL error) and BOC_c_(3,3) was the best in the multipath error performance at a typical multipath delay corresponding to urban areas.

**Figure 11:**
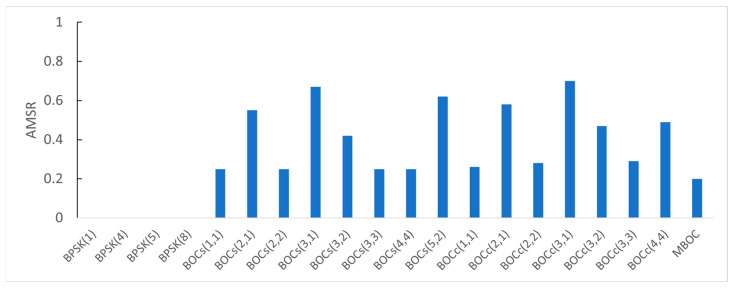
The AMSR for each candidate.

As seen in Figure 11, the AMSR of the BPSK signals was close to zero. BOC_s_(3,1) and BOC_c_(3,1), which had the highest modulation order, had high AMSRs of 0.67 and 0.70, respectively. Since a signal with a high AMSR requires a relatively complex receiver implementation, BPSK modulation schemes are preferred for civilian uses.

## 6. Conclusions

This paper presents signal performance analysis results for the possible S-band signals. In order to comprehensively evaluate the performance of the candidates, we considered the various signal performances. Each signal performance was analyzed using the FoM.

The analysis results showed that the outstanding candidate signal was different depending on the signal performance of interest and the reception environments. When we assumed the reception environments given in Table 6 and a multipath delay of 51 m, BPSK(1), BOC_s_(5,2), and BPSK(n) (n= 1, 4, 5, or 8) were superior for the spectral efficiency, ranging performance, and receiver complexity, respectively. The RF compatibility of the candidates was evaluated differently by the existing signal. As for the NavIC SPS and Globalstar, BOC_c_(4,4) was the best. Meanwhile, as for the NavIC RS signal, BOC_s_(3,1) was the best.

These results clearly showed that there was a trade-off relationship between the signal performances and that a comprehensive evaluation of candidates was an essential process. For example, candidates with good RF compatibility with the NavIC SPS signal and ranging performance tended to have poor spectral efficiency and receiver complexity. It implied that if the ranging performance is the primary requirement of the designed signal, we need to accept the power loss due to the OOBE, as well as the high receiver implementation costs.

The signal performance analysis methodology and results presented in this work can be used as a helpful reference for the S-band signal design for KPS. In future work, we will conduct a detailed signal performance analysis considering the trade-off relationship and weight of each signal performance according to the purpose of the service.

## Figures and Tables

**Table 1:** Frequency allocations in the S-band and adjacent bands.

Frequency (MHz)	Allocated Services
2450–2483.5	Fixed Mobile Radiolocation *
2483.5–2500	Fixed Mobile Mobile satellite (space-to-Earth) Radiolocation * RDSS (space-to-Earth)
2500–2520	Fixed Fixed satellite (space-to-Earth) ** Mobile (except aeronautical mobile) Mobile satellite (space-to-Earth) ***

* Secondary service in region 1. ** Not allocated in region 1. *** Only allocated in region 3.

**Table 2:** Signal design parameters of the S-band Navigation with Indian Constellation (NavIC) signals.

Service Name	Center Frequency (MHz)	Modulation	Code Length (Family)	Message Type	Data Rate
SPS-S	2492.028	BPSK(1)	1023 chips (Gold)	IRNSS	25 bps (50 sps)
RS-S		BOC_s_(5,2)	N/A	N/A	N/A

**Table 3:** Center frequency and modulation methods for each candidate.

Center Frequency (MHz)	Modulation Type	Chip Rate $f_{c}$ (Mcps)	Subcarrier Frequency $f_{s}$ (MHz)	Remarks
2492.028	BPSK-R	1.023	-	NavIC S-SPS [22]
		4.092	-	[22]
		5.115	-	-
		8.184	-	[22] [17]
	Sine-BOC (BOC_s_)	1.023	1.023	[22]
		1.023	2.046	-
		2.046	2.046	-
		1.023	3.069	-
		2.046	3.069	-
		3.069	3.069	-
		4.192	4.192	[23]
		2.046	5.115	NavIC S-RS
	Cosine-BOC (BOC_c_)	1.023	1.023	-
		1.023	2.046	-
		2.046	2.046	-
		1.023	3.069	-
		2.046	3.069	-
		3.069	3.069	-
		4.192	4.192	-
	MBOC	1.023	1.023 6.138	[22]

**Table 4:** Spectral separation coefficient (SSC) between existing signals and candidates: $B_{r}$ = 16.5 MHz or $B_{r}$ = $2f_{c}$ or 2($f_{s}+f_{c}$).

Candidates	$B_{r}$ (MHz)	SSC (dB/Hz)				$B_{r}$ (MHz)	SSC (dB/Hz)			
		BPSK(1) (S-SPS)	BOC_s_(5,2) (S-RS)	Globalstar (for 1 Ch.)	Self SSC		BPSK(1) (S-SPS)	BOC_s_(5,2) (S-RS)	Globalstar (for 1 Ch.)	Self SSC
BPSK(1)	16.5	−61.85	−77.80	−73.04	−61.85	2.046	−61.86	−77.51	−73.04	−61.86
BPSK(4)		−66.47	−78.27	−77.45	−67.85	8.184	−66.48	−79.80	−77.45	−67.86
BPSK(5)		−67.36	−78.76	−78.37	−68.81	10.23	−67.36	−78.95	−78.37	−68.82
BPSK(8)		−69.27	−74.86	−80.34	−70.83	16.368	−69.27	−74.86	−80.34	−70.83
BOC_s_(1,1)		−67.86	−80.04	−75.80	−64.84	4.092	−67.87	−83.33	−75.80	−64.88
BOC_s_(2,1)		−73.87	−76.66	−76.83	−66.06	6.138	−73.88	−77.90	−76.83	−66.11
BOC_s_(2,2)		−73.87	−77.81	−77.48	−67.83	8.184	−73.88	−79.67	−77.48	−67.86
BOC_s_(3,1)		−77.38	−80.34	−76.87	−66.35	8.184	−77.39	−80.88	−76.87	−66.35
BOC_s_(3,2)		−73.40	−77.33	−78.01	−68.72	10.23	−73.40	−77.34	−78.01	−68.73
BOC_s_(3,3)		−77.39	−76.83	−79.03	−69.59	12.276	−77.39	−76.83	−79.03	−69.59
BOC_s_(4,4)		−79.87	−72.97	−80.28	−70.82	16.368	−79.87	−72.97	−80.28	−70.82
BOC_s_(5,2)		−77.80	−69.20	−78.45	−69.20	14.322	−77.80	−69.20	−78.45	−69.20
BOC_c_(1,1)		−73.88	−79.37	−76.52	−66.08	4.092	−74.03	−84.27	−76.52	−66.28
BOC_c_(2,1)		−79.89	−75.29	−76.37	−66.43	6.138	−80.09	−78.02	−76.37	−66.53
BOC_c_(2,2)		−79.88	−74.87	−78.27	−69.06	8.184	−80.00	−79.40	−78.27	−69.24
BOC_c_(3,1)		−83.44	−77.85	−77.18	−66.53	8.184	−83.62	−80.68	−77.18	−66.54
BOC_c_(3,2)		−83.44	−74.83	−78.42	−69.37	10.23	−83.52	−75.28	−78.42	−69.42
BOC_c_(3,3)		−83.42	−72.87	−79.75	−70.84	12.276	−83.49	−72.88	−79.75	−70.95
BOC_c_(4,4)		−85.97	−71.17	−80.89	−72.16	16.368	−85.97	−71.17	−80.89	−72.16
MBOC		−68.26	−78.52	−76.20	−65.61	14.328	−68.26	−78.52	−76.20	−65.61

**Table 5:** Spectral efficiency of candidates in the S-band.

Candidates	B (MHz)	Spectral Efficiency
BPSK(1)	16.5	0.99
BPSK(4)		0.95
BPSK(5)		0.95
BPSK(8)		0.91
BOC_s_(1,1)		0.97
BOC_s_(2,1)		0.92
BOC_s_(2,2)		0.93
BOC_s_(3,1)		0.84
BOC_s_(3,2)		0.86
BOC_s_(3,3)		0.88
BOC_s_(4,4)		0.87
BOC_s_(5,2)		0.84
BOC_c_(1,1)		0.94
BOC_c_(2,1)		0.90
BOC_c_(2,2)		0.89
BOC_c_(3,1)		0.82
BOC_c_(3,2)		0.82
BOC_c_(3,3)		0.83
BOC_c_(4,4)		0.75
MBOC		0.95

**Table 6:** Receiver parameters used in the delay-locked-loop (DLL) error analysis (also used in the Gabor bandwidth and multipath error analysis).

T (s)	Tx. Filter		Rx. Filter		Code Tracking Parameters			
	Type	BW (MHz)	Type	BW (MHz)	$B_{L}$ (Hz)	Loop Order	Discriminator Type	EML Spacing (Chips)
0.02	-	∞	Brick-wall	16.5	0.1	1	NELP	0.1

**Table 7:** The DLL error and minimum required carrier-to-noise density ratio (*C*/*N*_0_) for stable DLL tracking of the candidates.

Candidates	DLL Error (m)		Minimum Required *C*/*N*_0_ (dB-Hz)
	*C*/*N*_0_ = 20 dB-Hz	*C*/*N*_0_ = 40 dB-Hz	
BPSK(1)	2.13	0.17	15.12
BPSK(4)	1.01	0.08	18.81
BPSK(5)	0.87	0.07	19.30
BPSK(8)	0.66	0.05	21.06
BOC_s_(1,1)	1.24	0.10	12.43
BOC_s_(2,1)	0.83	0.06	10.65
BOC_s_(2,2)	0.83	0.07	13.86
BOC_s_(3,1)	0.72	0.06	9.94
BOC_s_(3,2)	0.72	0.06	13.25
BOC_s_(3,3)	0.74	0.06	15.40
BOC_s_(4,4)	0.59	0.05	15.77
BOC_s_(5,2)	0.43	0.03	10.79
BOC_c_(1,1)	0.97	0.08	11.31
BOC_c_(2,1)	0.73	0.06	10.16
BOC_c_(2,2)	0.65	0.05	12.66
BOC_c_(3,1)	0.66	0.05	9.73
BOC_c_(3,2)	0.56	0.04	12.06
BOC_c_(3,3)	0.50	0.04	13.50
BOC_c_(4,4)	0.47	0.04	14.65
MBOC	1.24	0.10	11.09

**Table 8:** Gabor bandwidths of the candidates in the S-band.

Candidates	Gabor Bandwidth (MHz)		
	$B_{r}$ = 4 MHz	$B_{r}$ = 16.5 MHz	$B_{r}=2f_{c}$ or 2($f_{s}+f_{c}$)
BPSK(1)	0.46	0.93	0.33
BPSK(4)	0.90	1.85	1.31
BPSK(5)	0.88	2.19	1.63
BPSK(8)	0.76	2.62	2.62
BOC_s_(1,1)	0.80	1.60	0.80
BOC_s_(2,1)	1.16	2.45	1.72
BOC_s_(2,2)	1.10	2.26	1.60
BOC_s_(3,1)	0.35	2.70	2.64
BOC_s_(3,2)	0.56	2.71	2.53
BOC_s_(3,3)	0.82	2.65	2.40
BOC_s_(4,4)	0.59	3.21	3.21
BOC_s_(5,2)	0.27	4.41	4.38
BOC_c_(1,1)	1.03	2.06	1.03
BOC_c_(2,1)	0.93	2.78	1.84
BOC_c_(2,2)	0.78	2.93	2.07
BOC_c_(3,1)	0.15	3.11	2.70
BOC_c_(3,2)	0.28	3.58	2.86
BOC_c_(3,3)	0.37	3.93	3.11
BOC_c_(4,4)	0.20	4.17	4.16
MBOC	0.76	2.23	2.12

**Table 9:** Running average (RA) of the multipath error envelope (MPEE) of the candidates in the S-band.

Candidates	Max. MP Error (m)		RA of MPEE (m) ($B_{r}$ = 16.5 MHz)		
	$B_{r}=\infty$	$B_{r}$ = 16.5 MHz	$\tau_{delay}$ = 26 m	$\tau_{delay}$ = 51 m	$\tau_{delay}$ = 400 m
BPSK(1)	7.35	7.12	4.72	5.66	4.82
BPSK(4)	1.83	5.83	4.18	4.47	0.86
BPSK(5)	1.46	5.31	3.89	4.16	0.73
BPSK(8)	0.98	5.98	4.20	3.09	0.41
BOC_s_(1,1)	7.35	7.10	4.72	5.65	3.00
BOC_s_(2,1)	7.35	6.97	4.67	5.59	2.23
BOC_s_(2,2)	3.68	6.00	4.24	4.67	1.14
BOC_s_(3,1)	7.35	8.12	5.07	4.81	2.02
BOC_s_(3,2)	3.69	7.58	4.95	4.81	1.14
BOC_s_(3,3)	2.46	6.95	4.72	4.60	0.86
BOC_s_(4,4)	1.83	6.04	4.18	3.11	0.50
BOC_s_(5,2)	3.68	4.90	3.22	2.87	0.63
BOC_c_(1,1)	7.35	7.03	4.69	5.62	2.13
BOC_c_(2,1)	7.35	6.66	4.48	4.64	1.76
BOC_c_(2,2)	3.68	6.06	4.19	3.42	0.80
BOC_c_(3,1)	6.82	7.03	4.60	3.95	1.73
BOC_c_(3,2)	3.68	5.56	3.90	2.93	0.83
BOC_c_(3,3)	2.46	4.80	3.35	2.20	0.54
BOC_c_(4,4)	1.83	4.72	3.24	2.48	0.37
MBOC	6.18	5.32	3.41	3.18	1.66

**Table 10:** Autocorrelation main peak-to-secondary peak ratio (AMSR) of candidates in the S-band.

Candidates	AMSR		
	$B_{r}=\infty$	$B_{r}$ = 16.5 MHz	$B_{r}=2f_{c}$ or 2($f_{s}+f_{c}$)
BPSK (1)	0	0	0
BPSK(4)	0	0	0
BPSK(5)	0	0	0
BPSK(8)	0	0	0
BOC_s_(1,1)	0.25	0.25	0.25
BOC_s_(2,1)	0.56	0.55	0.54
BOC_s_(2,2)	0.25	0.25	0.25
BOC_s_(3,1)	0.68	0.67	0.68
BOC_s_(3,2)	0.44	0.42	0.42
BOC_s_(3,3)	0.25	0.25	0.25
BOC_s_(4,4)	0.25	0.25	0.25
BOC_s_(5,2)	0.62	0.62	0.62
BOC_c_(1,1)	0.25	0.26	0.49
BOC_c_(2,1)	0.56	0.58	0.77
BOC_c_(2,2)	0.25	0.28	0.49
BOC_c_(3,1)	0.68	0.70	0.87
BOC_c_(3,2)	0.44	0.47	0.67
BOC_c_(3,3)	0.25	0.29	0.49
BOC_c_(4,4)	0.25	0.49	0.49
MBOC	0.18	0.20	0.20

## Data Availability

Not applicable.
